# P-843. Inappropriate Antibiotic Prescribing for Acute Respiratory Illnesses in Outpatient Settings in New York City, 2019–2022

**DOI:** 10.1093/ofid/ofaf695.1051

**Published:** 2026-01-11

**Authors:** Celina Santiago, Katelynn Devinney, William Greendyke, Molly M Kratz, Elise Mantell, Elizabeth Cave, Karen Alroy, Nicole Burton

**Affiliations:** NYC Department of Health and Mental Hygiene, Queens, NY; NYC Department of Health and Mental Hygiene, Queens, NY; NYC Department of Health and Mental Hygiene, Queens, NY; NYC Department of Health and Mental Hygiene, Queens, NY; NYC Department of Health and Mental Hygiene, Queens, NY; N/A, London, England, United Kingdom; NYC Department of Health and Mental Hygiene, Queens, NY; NYC Department of Health and Mental Hygiene, Queens, NY

## Abstract

**Background:**

Inappropriate antibiotic prescribing contributes to antibiotic resistance. We assessed visit, patient, and provider attributes associated with inappropriate antibiotic prescribing for acute respiratory illnesses (ARI) at outpatient visits to New York City-based healthcare providers.

**Figure:**
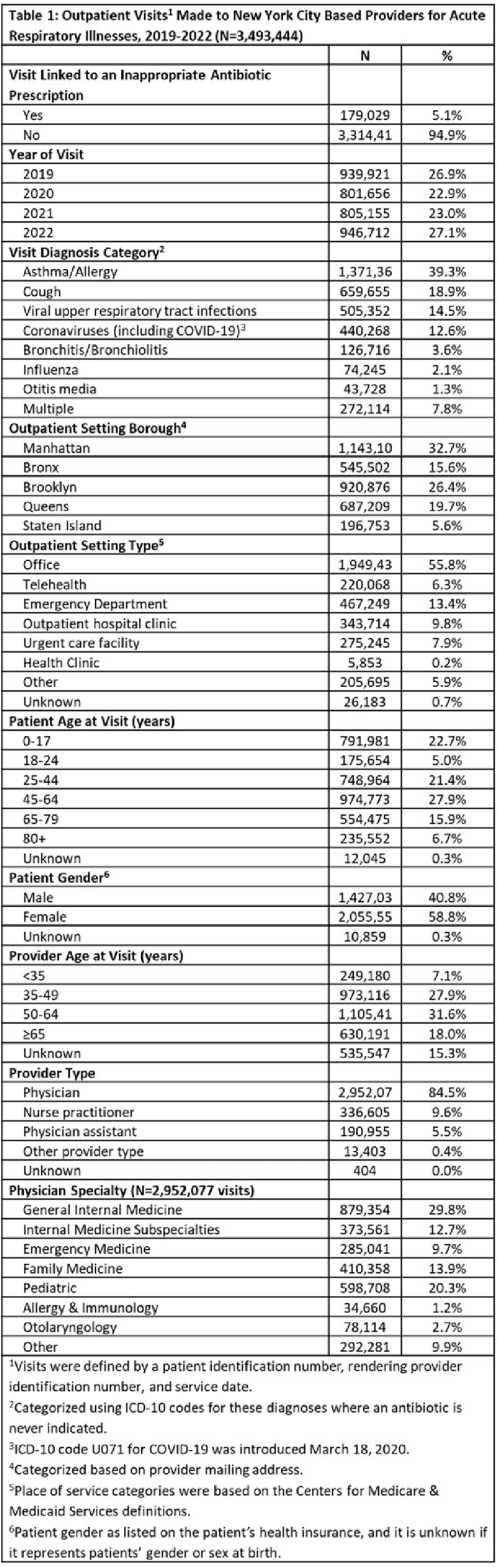

**Figure:**
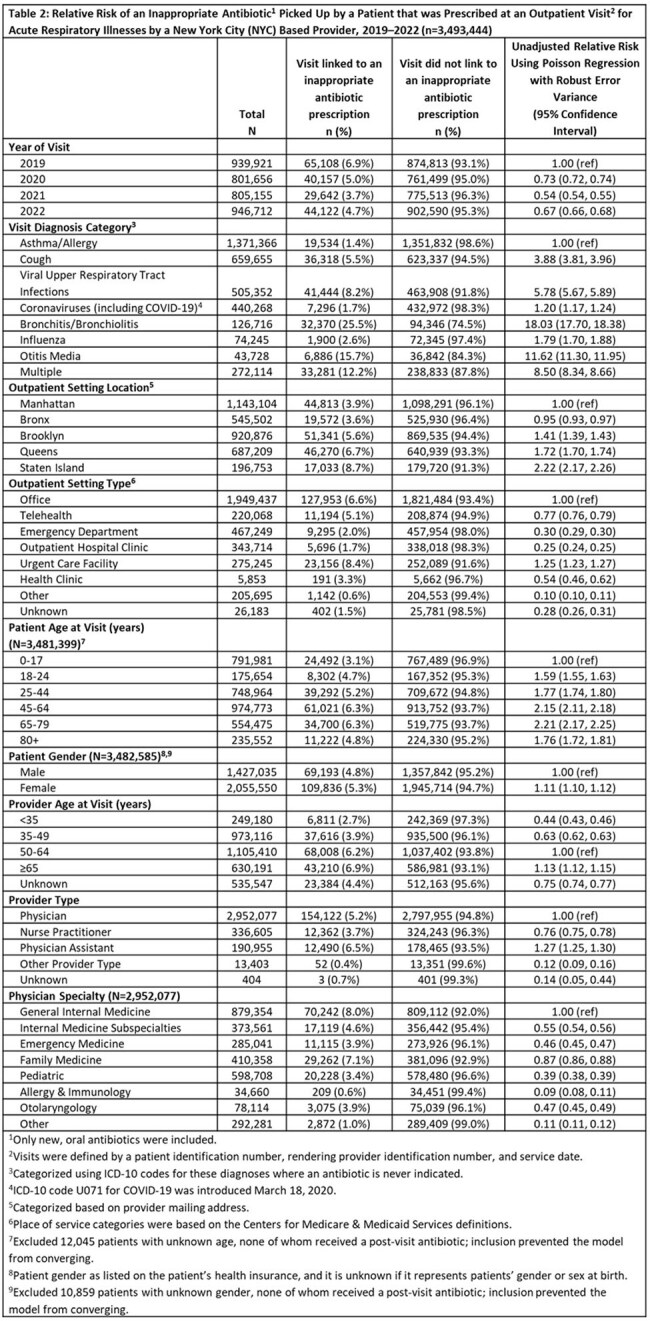

**Methods:**

We used the IQVIA Medical Claims dataset and ICD-10 codes to identify outpatient visits for ARI where antibiotics are never indicated (e.g., influenza, asthma) during 2019–2022. Visits were linked to claims from the IQVIA Longitudinal Prescription dataset for antibiotic prescriptions obtained at a pharmacy within 3 days post-visit. Univariate analyses were done to describe visit, patient, and provider attributes. Bivariate analyses using modified Poisson regression with robust error variance were used to calculate unadjusted relative risks (RR) and 95% confidence intervals (CI) for inappropriate prescribing.

**Results:**

Of 3,493,444 ARI outpatient visits, 5.1% linked to an inappropriate prescription (Table 1). Bronchitis/bronchiolitis visits were infrequent (3.6%) (Table 1) but had the highest percentage (25.5%) of inappropriate prescribing (Table 2). As shown in Table 2, compared with asthma/allergy visits, the RR of inappropriate prescribing was highest for bronchitis/bronchiolitis at 18.03 (95% CI: 17.70, 18.38) and lowest for coronaviruses at 1.20 (95% CI: 1.17, 1.24) and influenza at 1.79 (95% CI: 1.70, 1.88). By setting, inappropriate prescribing was highest in urgent care settings (8.4%), with a RR of 1.25 (95% CI: 1.23, 1.27) relative to office visits. By patient age, adults age 65-79 were more than twice as likely to be prescribed inappropriate antibiotics relative to pediatric patients (RR: 2.21; 95% CI: 2.17, 2.25). By provider type, inappropriate prescribing was highest for physician assistants (PAs) (6.5%), with a RR of 1.27 (95% CI: 1.25, 1.30) relative to physicians.

**Conclusion:**

Opportunities to improve inappropriate prescribing were identified. Diagnostic tests might improve prescribing practices, as visits for infections with point-of-care tests, such as influenza, had less inappropriate prescribing. We plan to reinforce prescribing education among providers (e.g. PAs) and settings (e.g. urgent care) with increased risk of inappropriate prescribing.

**Disclosures:**

All Authors: No reported disclosures

